# Supplementary figures and images for: Ustilago maydis Trf2 ensures genome stability by antagonizing Blm-mediated telomere recombination: Fine-tuning DNA repair factor activity at telomeres through opposing regulations
Source: PLoS Genet. 2024 Dec 9;20(12):e1011515. doi: 10.1371/journal.pgen.1011515 (PMC11670948; doi:10.1371/journal.pgen.1011515)

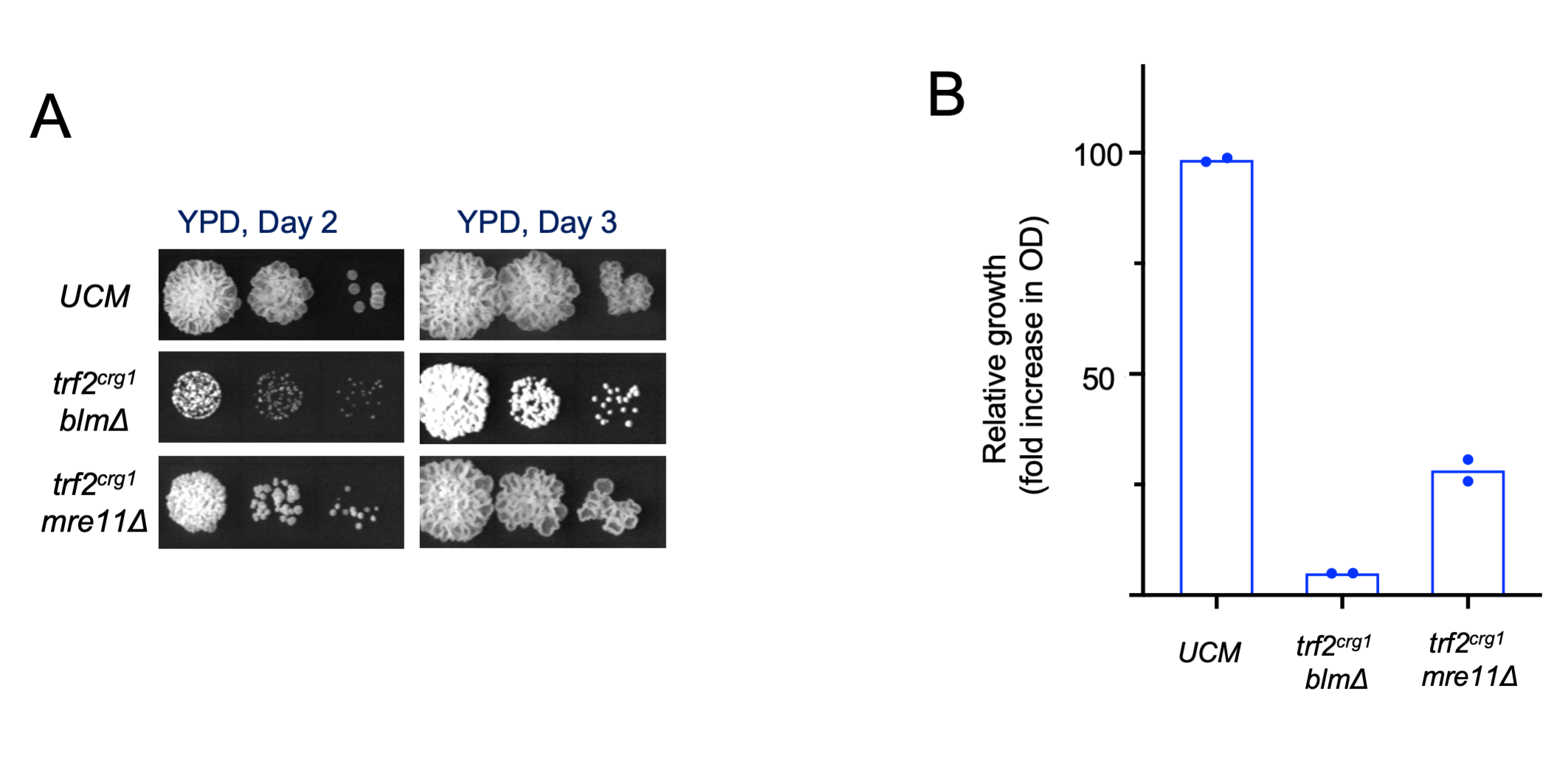

Supplement: S1 Fig — A. Serial dilutions of the indicated strains were spotted onto YPD medium. The growth of the strains was imaged after 2 days and 3 days. B. The indicated strains were inoculated into fresh YPD at an OD600 of 0.01 and grown at 30 degree for 17 hours. The fold increases in OD600 for two independent cultures were calculated and plotted. (TIF) [file pgen.1011515.s001.tif]

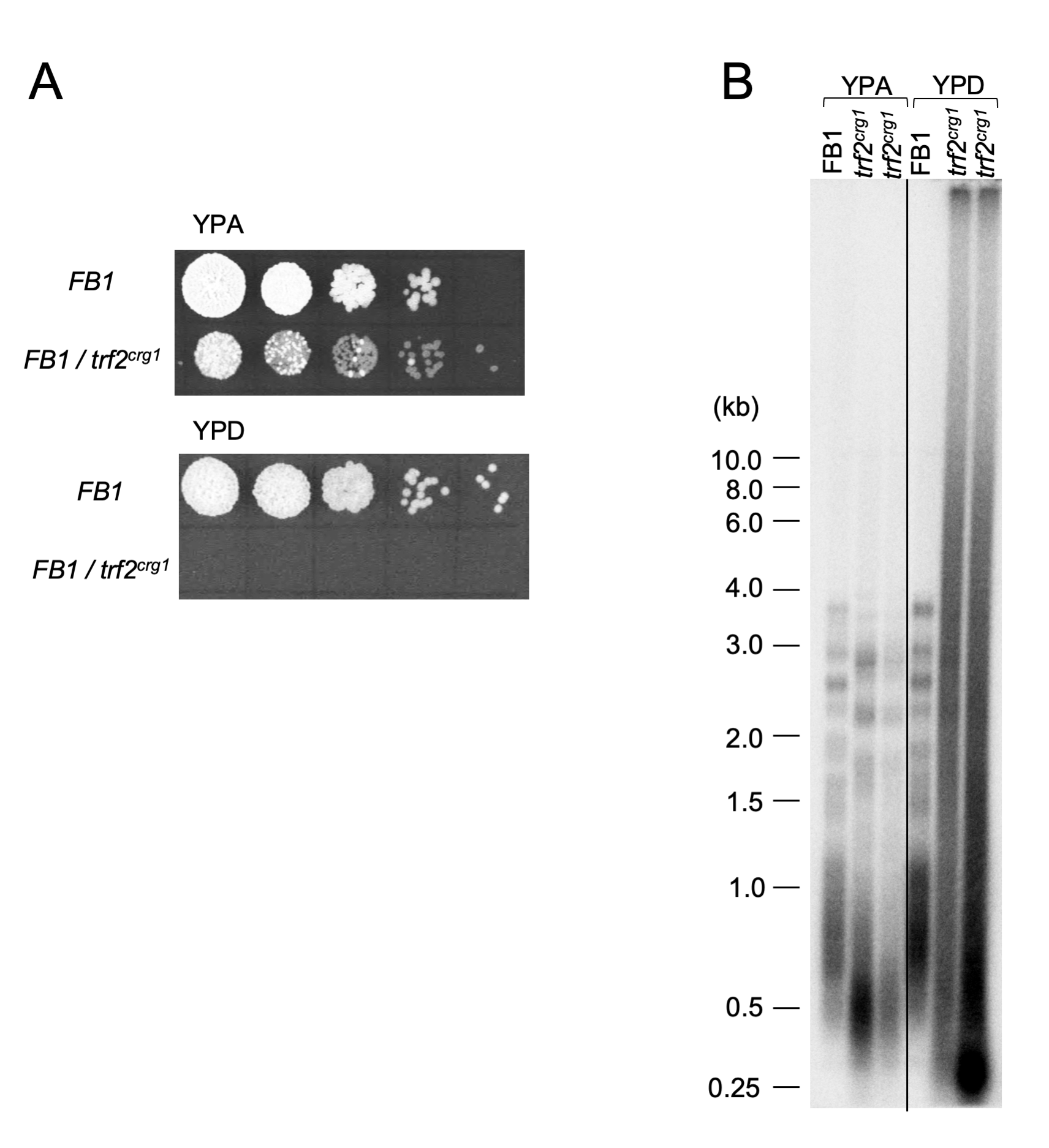

Supplement: S2 Fig — A. Serial dilutions of the indicated strains were spotted onto YPA and YPD media and incubated at 30°C. Following 2 days of growth, the plates were imaged. B. Genomic DNAs from the indicated strains were subjected to TRF Southern analysis. (TIF) [file pgen.1011515.s002.tif]

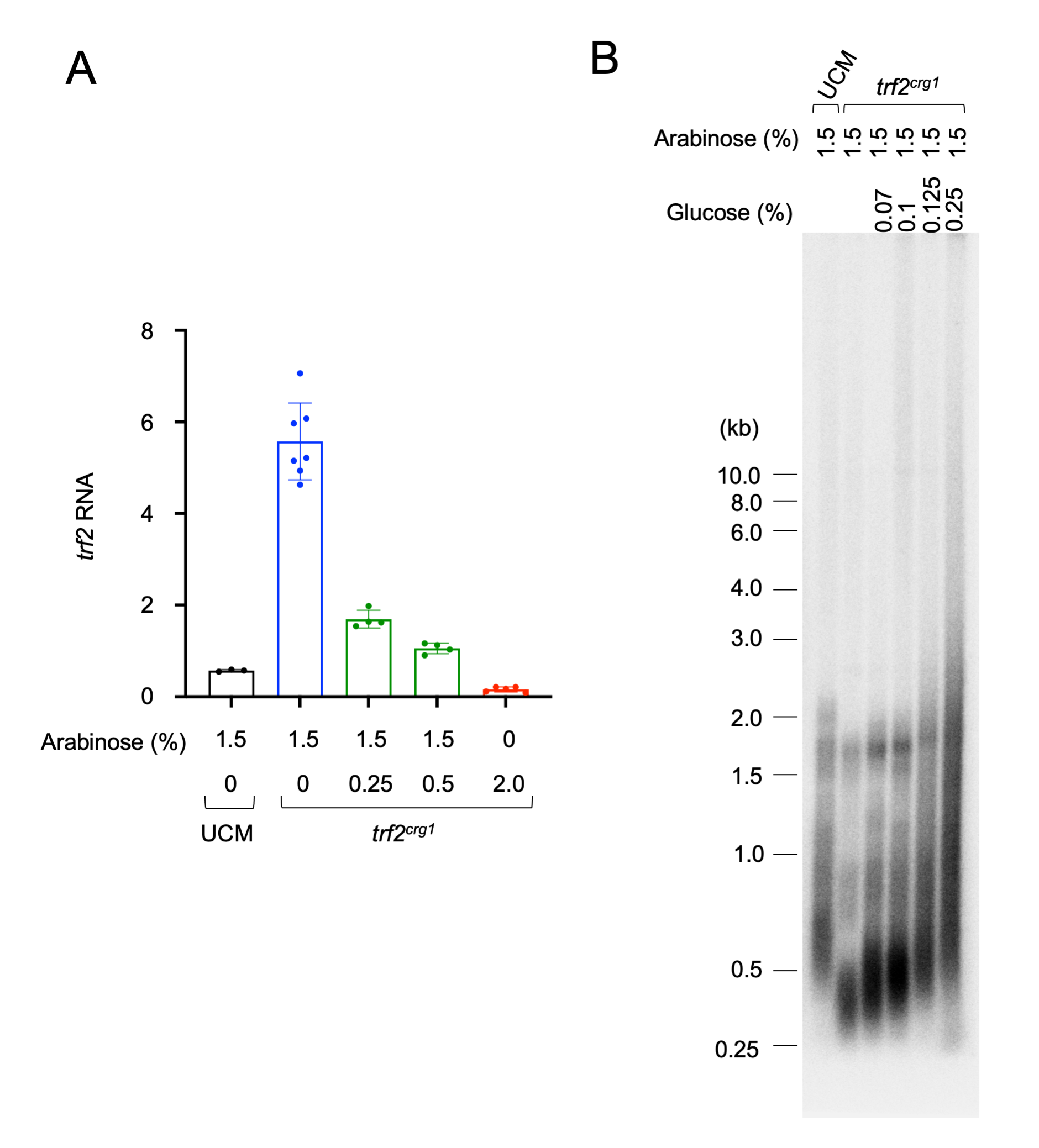

Supplement: S3 Fig — A. UCM and trf2crg1 were grown in media containing 1.5% arabinose and varying concentrations of glucose (to reduce the activity of the crg1 promoter). RNAs were isolated from these cultures and subjected to RT-qPCR analysis to determine the relative trf2 RNA levels. Data (mean ± SD) were derived from three or more independent experiments. B. Genomic DNAs were isolated from UCM and trf2crg1 grown in media with the specified arabinose and glucose concentrations and subjected to TRF Southern analysis. (TIF) [file pgen.1011515.s003.tif]

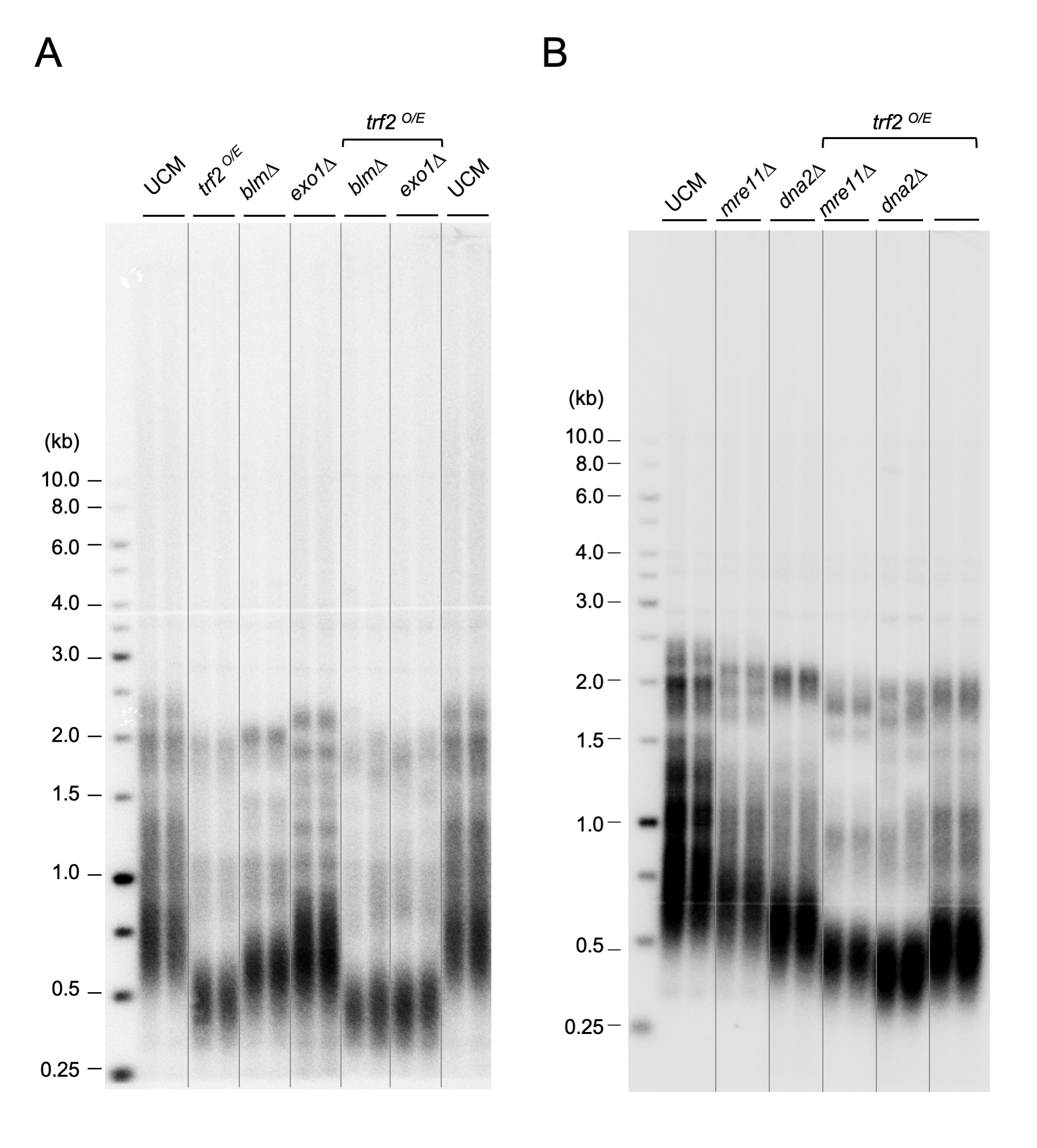

Supplement: S4 Fig — A and B. Genomic DNAs were isolated from the indicated strains grown in YPA, digested with PstI, and subjected to TRF Southern analysis. (TIF) [file pgen.1011515.s004.tif]

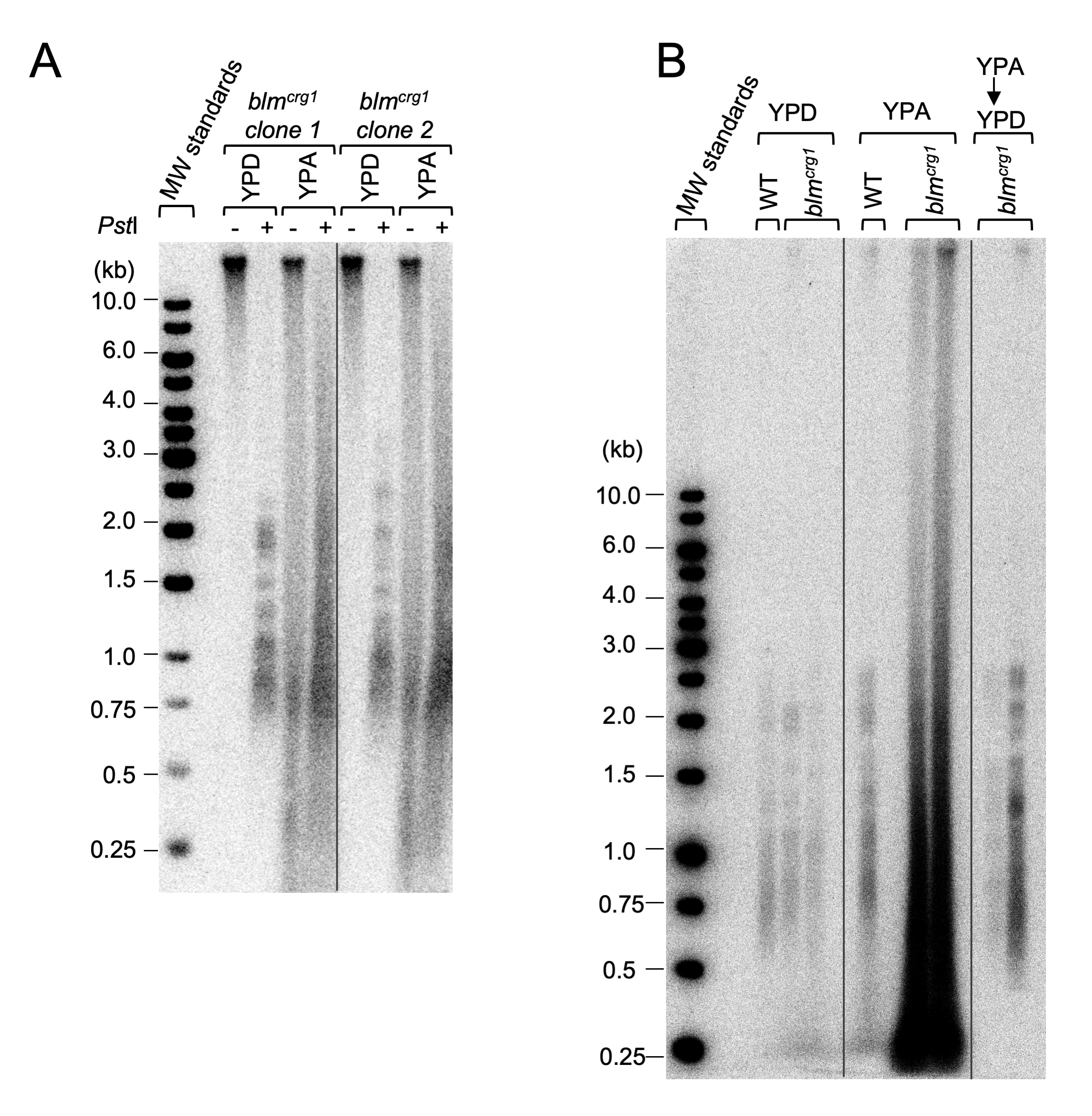

Supplement: S5 Fig — A. DNAs were isolated from blmcrg1 strains grown in the specified culture media and subjected to Southern analysis for telomere DNA with and without prior PstI digestion. B. DNAs were isolated from UCM and blmcrg1 strains grown in the designated culture media and subjected to Southern analysis. To test the reversibility of telomere defects induced by Blm overexpression, the blmcrg1 clones were first grown on a YPA plate and then re-streaked once on a YPD plate prior to liquid culture growth and genomic DNA isolation (designated by YPA -> YPD). (TIF) [file pgen.1011515.s005.tif]

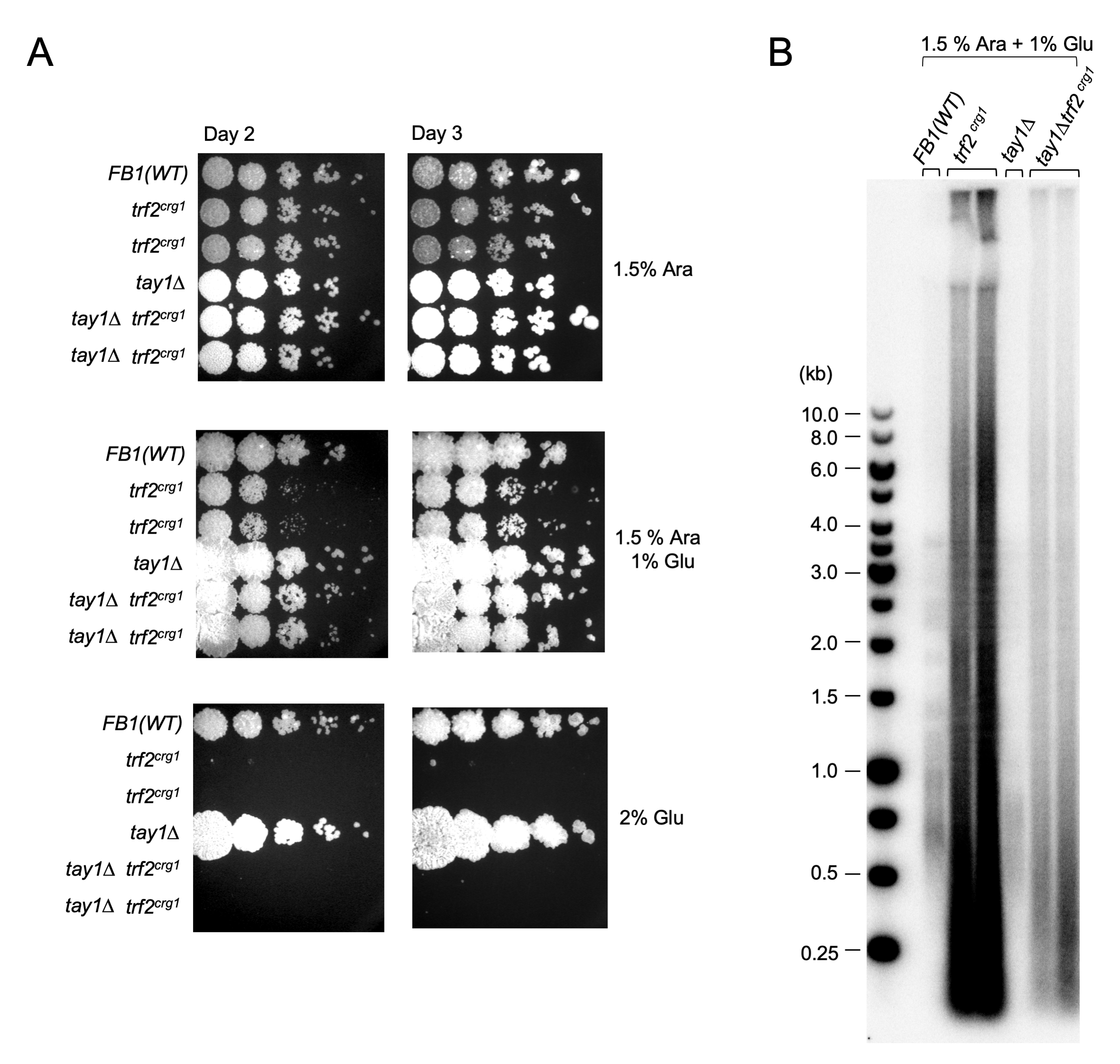

Supplement: S6 Fig — A. Serial dilutions of the designated strains were spotted onto semi-solid media containing the specified combinations of arabinose and glucose, and grown for 2 or 3 days at 30°C. B. Genomic DNAs were isolated from the indicated strains grown in 1.5% arabinose and 1% glucose, and subjected to TRF Southern analysis. (TIF) [file pgen.1011515.s006.tif]

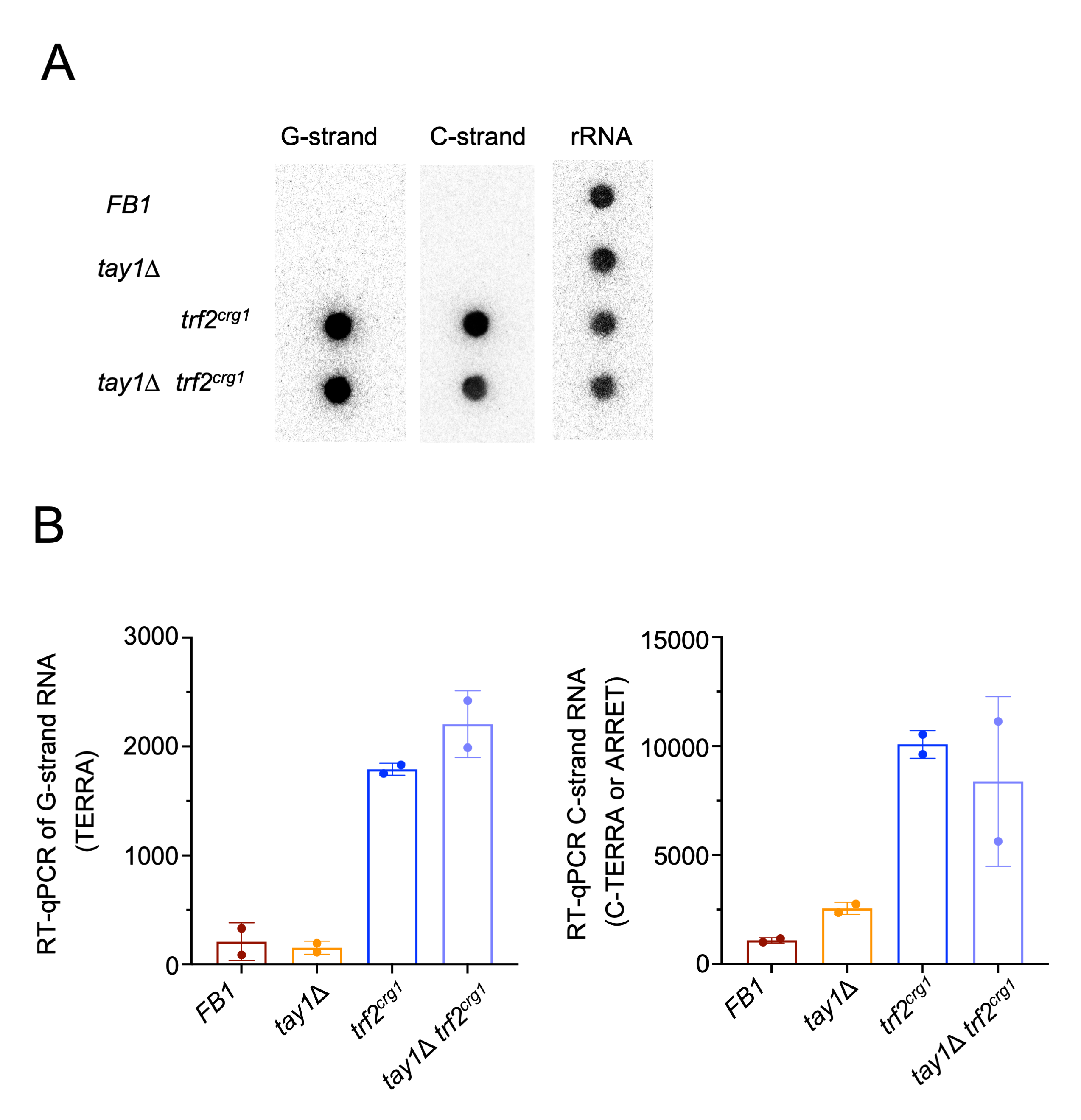

Supplement: S7 Fig — A. RNAs were isolated from the indicated strains grown in YPD and spotted onto nylon membrane. After crosslinking, the membrane was probed sequentially for G-strand RNA, C-strand RNA, and rRNAs. B. RNAs were isolated from the indicated strains grown in YPD, and subjected to RT-qPCR analysis to quantify the levels of G- and C-strand RNAs. For the G-strand RNA, the primers were designed to amplify TERRA (RNAs spanning subtelomeres and telomeres) from the UT6-bearing telomeres. For C-strand RNA, the primers can in principle amplify both C-TERRA (RNAs spanning subtelomeres and telomeres) and ARRET (RNAs with subtelomere sequences only) from UT6 telomeres. The quantities plotted (mean ± SD) represent cDNA copy numbers from 1 μl of RT reactions from two independent experiments. (TIF) [file pgen.1011515.s007.tif]
