# Supplementary material for: Ustilago maydis Trf2 ensures genome stability by antagonizing Blm-mediated telomere recombination: Fine-tuning DNA repair factor activity at telomeres through opposing regulations
Source: PLoS Genet. 2024 Dec 9;20(12):e1011515. doi: 10.1371/journal.pgen.1011515 (PMC11670948; doi:10.1371/journal.pgen.1011515)
Supplement: S1 Table — (DOCX) [file pgen.1011515.s008.docx]

**S1 Table. *U. maydis* strains used in this study**

| **Alias (Haploids)** | ***Relevant Genotype*** | **Reference** |
| --- | --- | --- |
| FB1^a^ | Wild type | Benuett and Herskowitz, 1989 [1] |
| USZ111 ^ab^ | *trf2 ^crg1^* | This work |
| UEY25 ^ac^ | *tay1∆* | Yu et al., 2020 [2] |
| USZ112 ^abc^ | *tay1∆ trf2 ^crg1^* | This work |
| UCM350 ^d^ | wild type | Kojic et al., 2002 [3] |
| USZ100 ^bd^ | *trf2 ^crg1^* | Yu et al., 2020 [2] |
| USZ121 ^bde^ | *blm∆ trf2^crg1^* | This work |
| USZ122 ^bdf^ | *dna2∆ trf2^crg1^* | This work |
| USZ123 ^bdg^ | *exo1∆ trf2^crg1^* | This work |
| USZ124 ^bdh^ | *mre11∆ trf2^crg1^* | This work |
| USZ125 ^bdi^ | *trf2^crg1^ rad51∆* | This work |
| USZ131 ^j^ | *blm^crg1^* | This work |
| USZ132 ^j^ | *blm^crg1^* | This work |
| USZ141 ^k^ | *blm^nar1^* | This work |
| USZ142 ^bk^ | *trf2^crg1^ blm^nar1^* | This work |
| USZ143 ^kl^ | *pot1^crg1^ blm^nar1^* | This work |

^a^ The genotype of FB1 is *a1b1* for the mating type loci.

^b^ *trf2* was placed downstream of the arabinose-dependent *crg1* promoter through the introduction of a Cbx^R^-containing cassette.

^c^ *tay1* was disrupted by the insertion of *hph* cassette expressing the hygromycin resistance gene *(Hyg^R^).*

^d^ The genotype of UCM350 *is nar1-6 pan1-1 a1b1*. *nar*, *pan*, and *ab* indicate inability to reduce nitrate, auxotrophic requirement for pantothenate, and mating type loci, respectively.

^e^ *blm* was disrupted by the insertion of *hph* cassette expressing the hygromycin resistance gene *(Hyg^R^).*

^f^ *dna2* was disrupted by the insertion of *hph* cassette expressing the hygromycin resistance gene *(Hyg^R^).*

^g^ *exo1* was disrupted by the insertion of *hph* cassette expressing the hygromycin resistance gene *(Hyg^R^).*

^h^ *mre11* was disrupted by the insertion of *hph* cassette expressing the hygromycin resistance gene *(Hyg^R^).*

^i^ *rad51* was disrupted by the insertion of *hph* cassette expressing the hygromycin resistance gene *(Hyg^R^).*

^j^ *blm* was placed downstream of the arabinose-dependent *crg1* promoter through the introduction of a Cbx^R^-containing cassette.

^k^ *blm* was placed downstream of the nitrate-dependent *nar1* promoter through the introduction of a Hyg^R^-containing cassette.

^l^ *pot1* was placed downstream of the arabinose-dependent *crg1* promoter through the introduction of a Cbx^R^-containing cassette.

**References:**

1. Banuett F, Herskowitz I. Different a alleles of Ustilago maydis are necessary for maintenance of filamentous growth but not for meiosis. Proceedings of the National Academy of Sciences of the United States of America. 1989;86(15):5878-82.

2. Yu EY, Zahid SS, Ganduri S, Sutherland JH, Hsu M, Holloman WK, Lue NF. Structurally distinct telomere-binding proteins in Ustilago maydis execute non-overlapping functions in telomere replication, recombination, and protection. Commun Biol. 2020;3(1):777.

3. Kojic M, Kostrub CF, Buchman AR, Holloman WK. BRCA2 homolog required for proficiency in DNA repair, recombination, and genome stability in Ustilago maydis. Mol Cell. 2002;10(3):683-91.
