# Supplementary material for: Ustilago maydis Trf2 ensures genome stability by antagonizing Blm-mediated telomere recombination: Fine-tuning DNA repair factor activity at telomeres through opposing regulations
Source: PLoS Genet. 2024 Dec 9;20(12):e1011515. doi: 10.1371/journal.pgen.1011515 (PMC11670948; doi:10.1371/journal.pgen.1011515)
Supplement: S2 Table — (DOCX) [file pgen.1011515.s009.docx]

**S2 Table. Oligonucleotides used in this study**

| **Name** | **Sequence 5’ to 3’** |
| --- | --- |
| **Protein Expression** |  |
| UmTrf2-416F-SalI | ATTAC GTCGAC CG CAA TCC GAA CAG CGG TTA |
| UmTrf2-dn-st-NotI | AA GCGGCCGC cta TTC GTT TGA AAG AGA ACT CGA CG |
| UmBlm-F-Nco | AAT CCATGG CA atg ccg caa tcc gca cta acc cca |
| UmBlm-R-FG-NotI | AAT GCGGCCGC cta CTT GTC ATC GTC ATC CTT GTA ATC acc cga acg agg tag att ggg c |
|  |  |
| **Strain Construction and Genotyping** |  |
| UmBlm(1)Nde | GAAATC CAT ATG CCG CAA TCC GCA CTA |
| UmBlm(720R)Xba | ATT TCTAGA CAA GAT CTG AAA CGA GCT CG |
| UmBlm(-720)Xba | ATA TCTAGA TTG GAA CTG CGC AGT AAG CT |
| UmBlm(-1R)Eco | ATT GAATTC AAC TGA AAG AAG GCA ATC CT |
| UmBlm(720R)Nsi | AGA ATGCAT CAA GAT CTG AAA CGA GCT CG |
| UmBlm(-720)Nsi | ATA ATGCAT TTG GAA CTG CGC AGT AAG CT |
|  |  |
| **Helicase assays** |  |
| NT – top | TTCTTCCTTTCCCTCTTCCTGATACGGCTGCTTCTCATCTACAACGTGATCCGTCATGGT |
| NT – bottom | ATGAGAAGCAGCCGTATCAGGAAGAGGGAAAGGAAGAA |
| Telo – top | TTCTTCCTTTCCCTC**TAGGGTTAGGGTTAGGGTTAGGGTTAGGGTTAGGGTTAGGGTTAG** |
| Telo – bottom | **CCCTAACCCTAACCCTAACCCTA**GAGGGAAAGGAAGAA |
|  |  |
| **PCR, hybridization, and EMSA assays** |  |
| TTAGGG_4_ (G4) | TTAGGG TTAGGG TTAGGG TTAGGG |
| CCCTAA_4_ (C4) | CCCTAA CCCTAA CCCTAA CCCTAA |
| TTAGGG_8_ (G8) | TTAGGG TTAGGG TTAGGG TTAGGG TTAGGG TTAGGG TTAGGG TTAGGG |
| CCCTAA_8_ (C8) | CCCTAA CCCTAA CCCTAA CCCTAA CCCTAA CCCTAA CCCTAA CCCTAA |
| UmrRNA_26S_121F | GCTTCGGACCATGCCTAAG |
| UmrRNA_26S_642R | CTTGGTCCGTGTTTCAAGACG |
|  |  |
| **RT-PCR and RT-qPCR** |  |
| UT6-TERRA-F1 | GGACGGCAGATATATATTGTGAGTGG |
| UT6-TERRA-F2 | GTGGCAACATTGGGTGAGC |
| UT6-TERRA-F2V2 | GATTTTGGTGGCAACATTGGGTGAGCA |
| UT6-TERRA-R1 | CCGTTGACACATTCAATCCCTC |
| UT6-TERRA-R2 | CTTCAAGCCCTGCAGCC |
| UT6-TERRA-R2V2 | ACAACTCTTCAAGCCCTGCAGCC |
| CCCTAA_4_ (C4) | CCCTAA CCCTAA CCCTAA CCCTAA |
| Blm-PCR-180F | cac tgc tca aaa gag atc gag gtc tgg |
| blm-PCR-209F | cat cgg ccg ctt cca tct caa acg |
| blm-PCR-305R | cga ggt gtc tgc gat atc gag cca c |
| Blm-PCR-400R | caa aat cga tcc gaa gct cgt ctt cg |
| Blm-PCR-500R | aag gct tcg tgc ttt tgg tac caa g |
| Trf2-PCR-1100R | TCT TCC GAG CCT TCA TCC GA |
| Trf2-PCR-865-F | CGC GAC ACC AAC CAT ACA AGC A |
| Trf2-PCR-939-R | GGT GAG TGG GGC ACT GTG TC |
|  |  |
| **STELA and fusion assays** |  |
| UT4-F | tcgggcaacgttccatgtcg |
| UT4-subtel-R2375 | Ccctcgaaggcagtgcatac |
| UT6-F | ctactacacatcggttcaggc |
| UT6-subtel-R2400 | atgccaaagtggaaatcgtgcac |
| C Telorette 1 | GCTCCGTGCATCTGGCATCCCCTAAC |
| C Telorette 2 | GCTCCGTGCATCTGGCATCTAACCCT |
| C Telorette 3 | GCTCCGTGCATCTGGCATCCCTAACC |
| C Telorette 4 | GCTCCGTGCATCTGGCATCCTAACCC |
| C Telorette 5 | GCTCCGTGCATCTGGCATCAACCCTA |
| C Telorette 6 | GCTCCGTGCATCTGGCATCACCCTAA |
| Teltail | GCTCCGTGCATCTGGCATC |
