# Supplementary material for: Ustilago maydis Trf2 ensures genome stability by antagonizing Blm-mediated telomere recombination: Fine-tuning DNA repair factor activity at telomeres through opposing regulations
Source: PLoS Genet. 2024 Dec 9;20(12):e1011515. doi: 10.1371/journal.pgen.1011515 (PMC11670948; doi:10.1371/journal.pgen.1011515)
Supplement: Attachment — Submitted filename: Response to Reviewers comments.pdf [file pgen.1011515.s012.pdf]

Response to Reviewers' comments

Reviewer #1: The article is best suited to an audience specialized in DNA replication and recombination at the end of chromosomes. This is a contribution to the knowledge of the mechanisms of maintaining telomere homeostasis.

Please also see the attachment.

In this article, the authors show the antagonistic control of UmTrf2 and Tay1 of Ustilago maydis on the activity of the Blm. The authors found that these telomere-binding proteins have different functions in telomere metabolism: UmTrf2 is essential, protects the chromosome end from NHEJ pathways of recombinational repair machinery, inhibits the Blm helicase activity, and has an epistatic relationship to it. Instead, Tay1 is not an essential protein in U. maydis; it plays a role in replication, promotes telomere recombination, and stimulates Blm activity. The deletion of UmTrf2 generates the accumulation of aberrant DNA forms due to an imbalance of the activities that generally occur at the chromosome end for its maintenance; such aberrant DNA production is alleviated by Blm and Mre11 deletion. Blm and Mre11 are needed for telomere replication and participate in the DNA resection of the chromosome end, indirectly controlling recombinational events. Antagonistic roles of UmTrf2 and Tay1 are demonstrated, as well as the finding of the previously reported TERRA and ARRET lncRNAs, but it was also found C-TERRA as a transcriptional product of dysfunctional chromosome ends in the designed experiments; notably, C-TERRA had not been identified previously except in the ascomycete Schizosaccharomyces pombe.

The work is splendid, sound, and well-founded, with original results showing features of the telomeric DNA protection mechanisms and how DDR by recombination repair systems operate in the basidiomycete U. maydis; this study also opens the possibility of investigating how telomere synthesis occurs or is controlled by lncRNAs under these circumstances.

We thank the reviewer for the positive assessment of this work.

The text is clear and focused; however, some minor details could be considered. In the numbered pages:

- Page 7. Last two rows: "However, subsequent analysis consistently revealed high levels of G-strand ssDNA, indicating that this is the reproducible phenotype of the mutant." Could this mean "also revealed a high..."
We believe the original results were incorrect, probably due to mislabeling of samples. We have modified the description to make it clear that the mutants accumulate high levels of G-strand ssDNA rather than C-strand ssDNA (page 7, last 3 lines).

- Page 9. Regarding exo1, "Additional analysis suggests that concerning telomere shortening, trf2O/E is also epistatic to exo1Δ, but not to dna2Δ (S4 Fig)." Please compare S4 Fig left vs. Fig.1B to avoid any matching problems.
We now point to the specific panels showing the epistasis relations between *trf2*$^{O/E}$ and *exo1Δ* (S4A Fig), and between *trf2*$^{O/E}$ and *dna2Δ* (S4B Fig) (page 9, first paragraph, bottom 3rd and 4th lines).

- Page 11. For a broader audience, please consider indicating what it is MMA.
We now indicate that MMA is a medium that contains both nitrate and arabinose, and can therefore support the expression from both the *nar1* and *crg1* promoters (page 12, first 2 lines).

- Page 22. In subsection Pull down and helicase assays... (Ref.) needs attention, please cite, or if you agree, please tell the audience what the Blm FLAG was in the main text.
The reference is provided (page 23, first line). The text has been modified to indicate the identities and locations of the tags on the Blm and Trf2$^{\Delta N}$ proteins used in the pull-down assays (page 22, bottom 2nd and 3rd lines; page 24, line 3-4).

- Page 47. S5A Fig. To my eyes, the legends from clone1 and clone2 growth on YPA and YPD were interchanged, as those results do not match those of S5B Fig and Fig 4C. In the same image, please consider moving the PstI legend away from the MWM lane or indicating the presence of the enzyme above the lanes of digested DNA to avoid confusion.
We have corrected the YPA/YPD designations in Fig. S5A, and moved the *Pst*I label away from the MW standards.

- Page 48. As FB1 was used throughout the experiment, it would be good to homogenize the legends (WT or FB1(WT)) of the first row of each panel in S6A Fig.
We now use FB1(WT) to label both the growth and Southern analysis in S6A and S6B.

- Page 36. In Fig. 7C, in the bottom right panels, please explain in the text what the red arrow is pointing at. Also, please explain why cDNA is amplified from transcripts from the UCM controls grown in YPD, as does in clone 2 (+ RT); please note that the same occurs even without reverse transcription (- RT) in the bottom right panel.
The arrow was accidentally moved from the TERRA illustration on the left side of Fig. 7C. This has been corrected. The UCM and blm^{crg1} #2 samples in TERRA panel displayed some RT independent PCR products. These are most likely due to DNA contamination and are explained in the figure legend (Fig. 7 legend, last 4 lines).

- Page 37. Please note the letters DDR are truncated in the image at the bottom left.
Corrected.

Reviewer #2:
In this manuscript, Zahid et al. examine the role of the telomere-binding protein Trf2 in repressing activity of the Blm helicase at telomeres in the fungal model organism Ustilago maydis. U. maydis telomeres are bound by Trf2, a protein orthologous to mammalian TRF2. Previous work has reported interactions between TRF2 and BLM helicase but the role of this interaction in telomere structure and function is unclear. Using genetic (knock-down and overexpression) studies and biochemical assays, the authors demonstrate that Trf2 negatively regulates Blm helicase and examine the opposing effects of telomere binding proteins Tay1 and Trf2 on Blm function at telomeres. The results contribute to the understanding of telomere biology and suggest that, at least in this organism, Blm repression is a critical function of Trf2. The genetic analysis would benefit from additional characterization of the conditional expression systems that the authors develop. Some additional information about statistical analyses is also required.

1. The conclusions of this manuscript are largely dependent on the conditional expression systems that are utilized. I recommend that the authors establish the extent of knock-down and overexpression up front (as a supplementary figure) prior to asking the reader to interpret the knock-down and overexpression phenotypes. Some data for trf2crg1 are included in Figure 3S where the extents of both expression changes are determined. Evidence of blm overexpression is shown in Figure 4A, but it is not clear how much expression is repressed on glucose. This is relevant because blm deletion causes telomere shortening (Figure 2A) but blmcrg1 on YPD has equivalent or even slightly longer telomeres than the WT strain. Why is this? The effect of the nar1 promoter on blm expression (used in Figure 5) should also be quantified and presented. In experiments where both blm and trf2 are under regulated control and the cells are grown on YPA, is blm expression repressed below normal levels?
We agree that it is important to validate the conditional expression systems utilized in this study. Because these conditional systems were utilized in different sections of the manuscript for different purposes, we find it easier to provide the expression data immediately before the phenotypic analysis of the relevant strains. Hence, in the revised manuscript, we provide the overexpression and repression data for the *trf2^{crg1}* allele in Fig 1A and Supp. Fig 3A. Data for the *blm^{crg1}* allele are shown in Fig. 4A, and data for the *blm^{nar1}* allele are shown in Fig. 5B.

In the original manuscript, the *blm*^{crg1} clones grown in YPD did not exhibit significant telomere shortening (as would be expected for Blm-deficient cells). This was most likely due to the fact that the strains were initially grown in YPA, and had been cultured in YPD for only about 20 generations before telomere analysis. We have repeated the analysis using *blm*^{crg1} cells that had been grown in YPD for ~60 generations (3 days) and have found that these cells do show significant telomere shortening (Fig. 4B).

For the experiments involving the *blm*^{nar1} allele, our analysis indicates that as predicted, *blm* RNA levels are elevated in the MMA medium and repressed in the YPA medium (for both the *blm*^{nar1} strain and the *blm*^{nar1} *trf2*^{crg1} strain) (Fig. 5B).

2. Where error bars are present, the figure legend or methods should indicate what those error bars represent. When statistical analysis is shown, the test used should be indicated. For example, is a test that accounts for multiple comparisons utilized in Figure 2B and C?I
We now indicate how the error bars are determined in each case (Legends for Fig. 1A, 2B, 2C, 4A, 4F, 5A and 7B; Supp. 3S and 7S). Student's t-test are used throughout the manuscript for pair-wise comparisons of samples and the calculation of p-values.

3. In cases where telomere lengths are analyzed in media that is non-permissive for colony formation, the length of time that cells have been grown in the indicated liquid medium should be stated.
The Trf2-deficient cells were grown for 16 to 24 hours in YPD prior to the analysis of telomere lengths and C-circles. This is now stated in the manuscript (page 22, bottom paragraph, line 1-3).

4. The first paragraph of the Discussion section entitled "The regulation of Blm by Trf2; functional connections between Trf2 and Ku70/Ku80 complex" needs to be better referenced with regards to the U. maydis results for Ku70 and Ku80. Those results aren't from this manuscript, are they? I don't see them anywhere but there are no references.
The references are now provided (page 15, second paragraph, line 4-6).

5. Is it possible to include molecular weight markers on the native and denatured gels (Figure 1D, for example) to allow the reader to ascertain how the sizes correlate to those in the standard Southern blots (Figure 1B, for example).
We now include markers for Figure 1D and 4E. The size resolution for the "in-gel" hybridization samples are limited because of the short durations of electrophoresis; the primary goal of the analysis was to determine the amount of single-stranded DNA rather than the size of DNA fragments with single-stranded DNA.

6. Figure 2B is lacking a Y axis label.
The label has been added.

7. In Figure 3A, "Pray" should be "Prey." Is Blm-FG really cross-reacting, or is there a fraction of the protein that wasn't cleaved during purification?
"Pray" has been corrected to "Prey". The lanes in the original Fig. 3A were mislabeled, making it hard to interpret the results. With the corrected labeling, the bands designated as Blm-FG are consistently and exclusively present in samples containing the Blm-FG bait and have the same mobility in the two blots (discussed in Fig. 3A legend). The species labeled His-SUMO-Trf2$^{\Delta N}$ is only detected in elution samples from the reaction containing both the bait and the prey, supporting our interpretation of specific pull-down.

8. Error bars should be included on Figure 3C. How was quantification done? This information should be added to the figure legend.
We performed the Blm helicase assays three times and consistently observed an inhibitory effect of Trf2 on telomeric DNA. The concentrations of Trf2 were somewhat different in different assays, so the data cannot be combined to generate error bars. This is now explained in the figure legend (Fig. 3C legend). We also added a description of the quantitation method; the signal for ssDNA was divided by the total signal in each lane to determine the fraction of DNA in the ssDNA form.

9. Figure 4E, F: Is 0 hours in YPA the correct comparison, especially for the G strand signal? It looks like repression of blm expression in YPD strongly increases G strand signal so that the change upon overexpression is being exaggerated compared to the change relative to normal expression levels. These results should be more carefully described and discussed.

The reviewer's point is well taken. Both the UCM control and *blm*^{crg1} clones grown in YPD exhibit more G-strand ssDNA than *blm*^{crg1} clones grown in YPA. We used the YPA 4 hr samples as the starting reference point of the plots because both Southern and C-circle analysis suggest that telomeres of the *blm*^{crg1} clones have not yet experienced high levels of recombination at this timepoint (Fig. 4C). We have included a more detailed discussion of the ssDNA analysis in the revised text (page 11, top paragraph, line 8-12).

10. In Figure 5SA, the YPA and YPD labels are switched.

The mistake has been corrected.
