# Supplementary material for: Ustilago maydis Trf2 ensures genome stability by antagonizing Blm-mediated telomere recombination: Fine-tuning DNA repair factor activity at telomeres through opposing regulations
Source: PLoS Genet. 2024 Dec 9;20(12):e1011515. doi: 10.1371/journal.pgen.1011515 (PMC11670948; doi:10.1371/journal.pgen.1011515)
Supplement: Attachment — Submitted filename: Response to Reviewers comments_2nd_revision.pdf [file pgen.1011515.s013.pdf]

**Response to Reviewers' comments**

**Reviewer #1:**
Authors of the article "Ustilago maydis Trf2 ensures genome stability by antagonizing Blm mediated telomere recombination: fine-tuning DNA repair factor activity at telomeres through opposing regulations" by Shahrez Syed, Sarah Aloe, Jeanette H. Sutherland, William K. Holloman, Neal F. Lue, have accomplished the suggestions that this reviewer made on the technical details and data presentation. The authors also fixed image troubles, clarified technical procedures, and improved the manuscript; hence, in the opinion of this reviewer, it satisfies the criteria for publication in the PLOS Genetics Journal.
The authors explore in an original approach the control of telomere maintenance and its sequence integrity by the interplay of Trf2 and helicase Blm with telomere repeats and possibly by a subtle interaction among those proteins to repress abnormal recombination events. In addition to the known involvement of Trf2 and Blm in telomere protection and replication/recombination, respectively, it is shown that severe imbalance in their appropriate expression rates causes aberrations in telomere sequence length and integrity, altered expression of ncRNAs from telomeric regions, loss of telomere homeostasis, and chromosome instability.

We thank the reviewer for the positive assessment of this work.

**Reviewer #2:**
The authors have addressed most of my concerns and I appreciate the clarifications added to the manuscript. My one remaining concern relates to the multiple statistical comparisons that are done in Figures 2 and 7. It is not appropriate to conduct t-tests when the same sample is compared to multiple other samples in the same experiment. ANOVA with a post-hoc test would correct for multiple comparisons. For example, see https://jbcresources.asbmb.org/collecting-and-presenting-data.

The reviewer makes a valid point. We have re-analyzed the data using ANOVA with Dunnett's multiple comparisons test and displayed the resulting p-values in Figures 2 and 7. This re-analysis has no impact on any of the conclusions.
